# CINSARC in high‐risk soft tissue sarcoma patients treated with neoadjuvant chemotherapy: Results from the ISG‐STS 1001 study

**DOI:** 10.1002/cam4.5015

**Published:** 2022-07-18

**Authors:** Anna Maria Frezza, Silvia Stacchiotti, Frederic Chibon, Jean‐Michelle Coindre, Antoine Italiano, Cleofe Romagnosa, Silvia Bagué, Angelo Paolo Dei Tos, Luca Braglia, Emanuela Palmerini, Vittorio Quagliuolo, Javier Martin Broto, Antonio Lopez Pousa, Giovanni Grignani, Antonella Brunello, Jean‐Yves Blay, Robert Diaz Beveridge, Iwona Lugowska, Tom Lesluyes, Roberta Maestro, Franco Domenico Merlo, Paolo Giovanni Casali, Alessandro Gronchi

**Affiliations:** ^1^ Department of Medical Oncology Fondazione IRCCS Istituto Nazionale Tumori Milano Italy; ^2^ Institut Claudius Régaud, Cancer Research Center of Toulouse (CRCT) IUCT‐ Oncopole Toulouse France; ^3^ Department of Biopathology Institut Bergonié Bordeaux France; ^4^ Early Phase Trials and Sarcoma Units Institut Bergonié Bordeaux France; ^5^ Clinical Genetics and Genetic Counseling Program Germans Trias i Pujol Hospital Barcelona Spain; ^6^ Department of Pathology Hospital de la Santa Creu i Sant Pau Barcelona Spain; ^7^ Department of Medicine University of Padua School of Medicine Padua Italy; ^8^ Department Infrastructure Research and Statistics Azienda USL‐IRCCS Reggio Emilia Reggio Emilia Italy; ^9^ Osteoncology, Bone and Soft Tissue Sarcomas and Innovative Therapies IRCCS Istituto Ortopedico Rizzoli Bologna Italy; ^10^ Sarcoma, Melanoma and Rare Tumors Surgery Unit IRCCS Humanitas Research Hospital Milan Italy; ^11^ Medical Oncology Department, University Hospital Fundación Jimenez Diaz, Madrid, Spain University Hospital General de Villalba, Madrid, Spain. Instituto de Investigacion Sanitaria Fundacion Jimenez Diaz (IIS/FJD; UAM) Madrid Spain; ^12^ Fundacio de Gestio Sanitaria de L'Hospital de la Santa Creu I Sant Pau Barcelona Spain; ^13^ Division of Medical Oncology, Candiolo Cancer Institute FPO – IRCCS Candiolo Italy; ^14^ Medical Oncology Istituto Oncologico Veneto Padova Italy; ^15^ Department of Medicine, Centre Leon Berard UNICANCER & University Lyon I Lyon France; ^16^ Department of Cancer Medicine Hospital Universitari i Politècnic La Fe Valencia Spain; ^17^ Department of Soft Tissue/Bone Sarcoma and Melanoma, Centrum Onkologii Instytut im. Marii Sklodowskiej‐Curie Warsaw Poland; ^18^ Oncogenetics and Oncogenomics Unit Centro di Riferimento Oncologico di Aviano IRCCS Aviano Italy; ^19^ Scientific Department Azienda USL‐IRCCS Reggio Emilia Reggio Emilia Italy; ^20^ Department of Surgery Fondazione IRCCS Istituto Nazionale dei Tumori Milan Italy

**Keywords:** chemotherapy, CINSARC, outcome, prognostication, sarcoma

## Abstract

**Background:**

The Complexity INdex in SARComas (CINSARC) is a transcriptional signature derived from the expression of 67 genes involved in mitosis control and chromosome integrity. This study aims to assess CINSARC value of in an independent series of high‐risk patients with localized soft tissue sarcoma (STS) treated with preoperative chemotherapy within a prospective, randomized, phase III study (ISG‐STS 1001).

**Patients and Methods:**

Patients with available pre‐treatment samples, treated with 3 cycles of either standard (ST) preoperative or histotype‐tailored (HT) chemotherapy, were scored according to CINSARC (low‐risk, C1; high‐risk, C2). The 10‐year overall survival probability (pr‐OS) according to SARCULATOR was calculated, and patients were classified accordingly (low‐risk, Sarc‐LR, 10‐year pr‐OS>60%; high‐risk, Sarc‐HR, 10‐year pr‐OS<60%). Survival functions were estimated using the Kaplan–Meier method and compared using log‐rank test.

**Results:**

Eighty‐six patients were included, 30 C1 and 56 C2, 49 Sarc‐LR and 37 Sarc‐HR. A low level of agreement between CINSARC and SARCULATOR was observed (Cohen's Kappa = 0.174). The 5‐year relapse‐free survival in C1 and C2 were 0.57 and 0.55 (*p* = 0.481); 5‐year metastases‐free survival 0.63 and 0.64 (*p* = 0.740); 5‐year OS 0.80 and 0.72 (*p* = 0.460). The 5‐year OS in C1 treated with ST and HT chemotherapy was 0.84 and 0.76 (*p* = 0.251) respectively; in C2 treated it was 0.72 and 0.70 (*p* = 0.349). The 5‐year OS in Sarc‐LR treated with S and HT chemotherapy was 0.80 and 0.82 (*p* = 0.502) respectively; in Sarc‐HR it was 0.70 and 0.61 (*p* = 0.233).

**Conclusions:**

Our results, although constrained by the small size of the series, suggest that CINSARC has weak prognostic power in high‐risk, localized STS treated with neoadjuvant chemotherapy.

## Graphical abstract

This study aims to assess the value of CINSARC, a transcriptional signature derived from the expression of 67 genes involved in mitosis control and chromosome integrity, in an independent series of high‐risk patients with localized soft tissue sarcoma (STS) treated with preoperative chemotherapy within a prospective, randomized, phase III study (ISG‐STS 1001). As it can be seen in the figure, which shows Kaplan Meier curves for relapse‐free survival, metastases‐free survival, and overall survival in CINSARC low‐risk (C1) and high‐risk (C2) patients, our results, although constrained by the small size of the series, suggest that CINSARC has a weak prognostic power in this population.

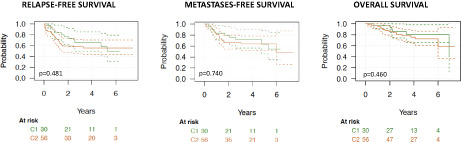

## 1 INTRODUCTION

The Complexity INdex in SARComas (CINSARC) is a transcriptional signature derived from the expression of 67 genes involved in mitosis control and chromosome integrity. Originally developed in 2010 by Chibon et al. on fresh‐frozen specimens analyzed by microarray, CINSARC has been subsequently implemented on archival FFPE samples for both RNA sequencing and NanoString hybridization platform (NanoCind®).^1^, ^2^

CINSARC has been claimed to outperform the histology‐based grading system of the *Fédération Nationale des Centres de Lutte Contre le Cancer* (FNCLCC), currently used for grading most types of soft tissue sarcoma (STS) types, based on the reportedly superior capacity to identify patients with a worse prognosis.^3^ It was reported as a predictor of metastatic outcome in STS patients, as a whole,^1^, ^3^ and particularly in patients with uterine leiomyosarcoma,^4^ smooth muscle tumor of unknown malignant potential (STUMP),^5^ synovial sarcomas^6^ and rhabdomyosarcoma of pediatric and adult ages as well as in gastrointestinal stromal tumors (GIST).^7^, ^8^ Furthermore, its prognostic value was also suggested in a broad range of other cancer types such as breast carcinomas and lymphomas.^9^, ^10^

On this basis, CINSARC was proposed as a potentially valuable tool to identify, among patients with localized STS, those at high‐risk who might benefit from pre‐operative chemotherapy, regardless of FNCLCC grade. A prospective, randomized, phase 3 trial aiming to explore the potential benefit of chemotherapy in high‐risk CINSARC patients and to prospectively validate the prognostic role of CINSARC in FNCLCC grade 1 and 2 STS has been designed by the French Sarcoma Group and it is due soon.^11^, ^12^

As of today, however, the value of CINSARC as a predictor of metastatic outcome in STS patients has been only explored in retrospective case series. In this study, we aim to explore the value of this signature in an independent series of high‐risk patients (high‐malignancy grade, 5 cm or larger in diameter, deeply located according to the investing fascia) with localized STS treated with preoperative chemotherapy within a prospective, randomized, phase III study (ISG‐STS 1001 study).^13^ Also, we assessed the concordance between CINSARC and SARCULATOR, a prognostic nomogram based on clinical‐pathological data (patient age, sarcoma type, FNCLCC grade and size), which has been tested and validated in large patient series and currently used in the everyday clinical practice.^14^, ^15^ The possible prognostic value of CINSARC across subgroups with a different survival probability, stratified through SARCULATOR, was explored.

## 2 PATIENTS AND METHODS

This study relies on the tumor series of the prospective, randomized, phase III ISG‐STS 1001 study involving patients affected by localized, high‐risk (high‐malignancy grade, 5 cm or larger in diameter, deeply located according to the investing fascia), STS of the extremities or trunk wall and treated with 3 cycles of either full‐dose standard (ST) pre‐operative chemotherapy with epirubicin plus ifosfamide or histotype‐tailored (HT) chemotherapy (trabectedin for high‐grade myxoid liposarcoma, gemcitabine and dacarbazine for leiomyosarcoma, high‐dose ifosfamide for synovial sarcoma, etoposide and ifosfamide for malignant peripheral nerve sheath tumor). In the context of the ISG‐STS 1001 study, the pathological diagnosis and grading of all included cases was centrally reviewed at the sarcoma reference centers contributing to the study, before random assignment. Cases for which pre‐treatment tumor samples were available for molecular analyses were included in the present series.^13^ The data that support the findings of this study are available on request from the corresponding author. The data are not publicly available due to privacy or ethical restrictions. The ISG‐STS 1001 study was approved by the institutional review board or ethics committee of each contributing institution and all patients included in the study signed a dedicated informed consent.

### 2.1 CINSARC scoring with the Nanocing technology

RNA was extracted from pretreatment samples for which FFPE material was available using the High Pure FFPET RNA Isolation Kit (Roche). Samples for which FFPE material was insufficient or unsuitable for molecular analysis were excluded from the analysis. Overall 86 cases were tested for CINSARC on a Nanostring platform. To assess CINSARC prognostic groups, we used NanoString data performed in Le Guellec et al. as a reference dataset of 94 cases strongly associated to either C1 or C2 group (good and poor prognosis groups, respectively).^2^ For each case, we normalized the 95 cases (94 from the reference dataset plus one single sample to test) altogether using an identical methodology as in the NanoString Solver software.^2^ After normalization, the nearest centroid method was used for sample clustering into C1 or C2 CINSARC risk‐group, using the dataset of Le Guellec et al.^2^ as a reference.

### 2.2 SARCULATOR prediction

Data of each patient included in the present series were analyzed using SARCULATOR, a prognostic nomogram for primary extremity STS (http://www.sarculator.com). SARCULATOR allows the prediction of 5‐ and 10‐year OS probability (pr‐OS) thanks to the integration of patient and tumor features: patient age (18–100 years), tumor size (0.1–35 cm), FNCLCC grade (I, II, and III), and tumor histology (myxoid liposarcoma, leiomyosarcoma, synovial sarcoma, malignant peripheral nerve sheet tumor, and undifferentiated pleomorphic sarcoma).^14^ Patients were then classified into 2 subgroups: low‐risk (Sarc‐LR, 10‐year pr‐OS 60%) and high‐risk (Sarc‐HR, 10 year pr‐OS<60%) risk. These survival subgroups were identified and proved to be predictive of a benefit from ST neo‐adjuvant chemotherapy in a previousl analysis performed on the whole ISG‐STS 1001 study population.^15^

### 2.3 Statistical analysis

In absence of an a‐priori hypothesis, given the exploratory nature of the study, no formal sample size calculation was performed.

Proportions between groups were compared using the chi‐square test or the Fisher's exact test if needed. Agreement between qualitative measurements was assessed by Cohen's kappa.^16^

Median follow‐up time was evaluated using the “reverse Kaplan–Meier” method on Overall Survival (OS).^17^ Survival functions were estimated using the Kaplan–Meier method and compared using log rank test. Point estimates at 3 and 5 years were reported for descriptive purposes.

OS time was measured from enrollment until death. Survival time of patients who did not experienced the event considered during follow up observation was censored at the time of the last follow up. Relapse Free Survival (RFS) was defined as time to local recurrence, distant metastasis or death (whichever comes first), censoring for last follow up. Metastasis Free Survival (MFS), was defined as time to distant metastasis or death (whichever comes first), censoring for local recurrence or last follow up.

Unless otherwise specified, confidence intervals were two‐tailed and calculated considering a 0.95 confidence level. Performed tests were considered statistically significant whether the *p*‐values were <0.05. Statistical analysis was performed using R 3.5.2.^18^

## 3 RESULTS

### 3.1 Population characteristics

Four hundred and thirty‐five patients with a primary localized high‐risk STS were included in the ISG‐STS 1001 study. Among them, 287 were randomized to either receive ST or HT neoadjuvant chemotherapy.^13^ Material suitable for the evaluation of the CINSARC signature was available for 86 pre‐treatment samples (56 and 30 treated with ST and HT neoadjuvant chemotherapy, respectively), which represent the cohort of this study. No treatment‐related deaths were observed in this group of patients. The median age of the patients was 52 years, with a male/female ratio of 53/33. According to NanoCind testing, 30 (35%) patients were classified as low‐risk (C1) and 56 (65%) as high‐risk (C2). By applying SARCULATOR to the same population, 49 (57%) patients scored as Sarc‐LR, whereas 37 (43%) as Sarc‐HR. Among Sarc‐LR patients, 21 were classified as C1 per CINSARC, 28 as C2; among Sarc‐HR, 9 were C1 and 28 C2. A low level of agreement between CINSARC and SARCULATOR was observed (Cohen's Kappa = 0.174; 95% CI: −0.012 to 0.360).

With a median follow‐up of 4.3 years, 13 (15%) patients developed local recurrence (LR) and 24 (28%) distant metastases (DMs). The median (m‐) OS in the whole population was 6.9 years. Population characteristics are detailed in Table 1.

**TABLE 1:** Population characteristics

	C1 (*N* = 30)	C2 (*N* = 56)
Median age (years)	49	52
Gender (M/F)	21/9	32/24
Primary site		
Trunk	1	6
Upper limbs/girdles	3	9
Lower limbs/girdles	26	41
Median primary size (mm)	84	99
Histology		
Myxoid‐round cell liposarcoma	12	0
Synovial sarcoma	9	4
Malignant peripheral nerve sheath tumor	2	8
Leiomyosarcoma	0	7
Undifferentiated pleomorphic sarcoma	4	19
Myxofibrosarcoma	3	11
Unclassified spindle cell	0	1
Pleomorphic liposarcoma	0	5
Pleomorphic rabdomiosarcoma	0	1
FNCLCC Grade		
2	6	2
3	24	54
Regimen		
Standard chemotherapy	17	39
Histology‐tailored chemotherapy	13	17
Radiation therapy (pre‐ or post‐operative)	20	45

### 3.2 CINSARC prognostic value: relapse‐free survival (RFS), metastases‐free survival (MFS) and overall survival (OS)

**FIGURE 1:**
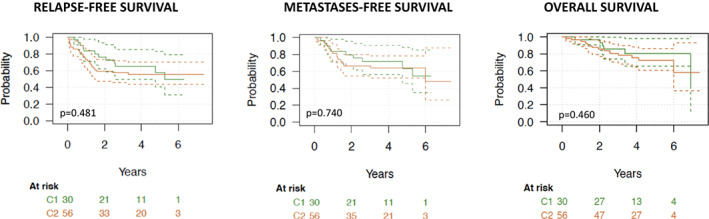
Kaplan Meier curves for RFS, MFS, and OS in CINSARC C1 and C2 patients.

The incidence of LRs and DMs in C1 and C2 patients was 2 (17%) versus 11 (44%) and 10 (83%) versus 14 (56%) respectively (*p* = 0.15). Overall, the 3‐ and 5‐year RFS in group C1 and C2 were 0.64 versus 0.57 and 0.57 versus 0.55 respectively (*p* = 0.481); 3 and 5‐year MFS in group C1 and C2 were 0.71 versus 0.63 and 0.66 versus 0.64 respectively (*p* = 0.740); 3‐ and 5‐year OS were 0.85 versus 0.80 and 0.80 versus 0.72 respectively (*p* = 0.460). Kaplan Meier curves for RFS, MFS, and OS are reported in Figure 1.

In the subgroup of patients treated with ST chemotherapy (*n* = 56), the 3‐ and 5‐year RFS in C1 and C2 were 0.60 versus 0.50 and 0.56 versus 0.53 respectively (*p* = 0.623); 3 and 5‐year MFS in group C1 and C2 were 0.73 versus 0.61 and 0.67 versus 0.63 respectively (*p* = 0.696); 3‐ and 5‐year OS were 0.93 versus 0.84 and 0.84 versus 0.72 respectively (*p* = 0.391).

**FIGURE 2:**
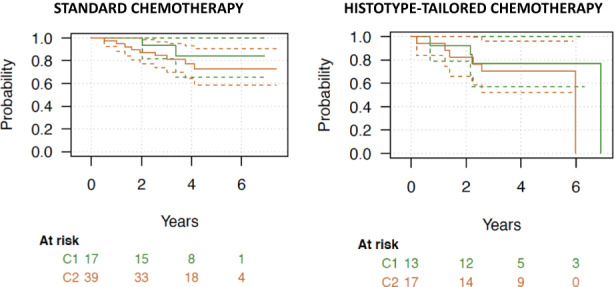
Kaplan Meier curves for OS by CINSARC prediction and treatment arm.

In the subgroup of patients treated with HT chemotherapy (*n* = 30), the 3‐ and 5‐year RFS in C1 and C2 were 0.69 versus 0.69 and 0.58 versus 0.58 respectively (*p* = 0.647); 3 and 5‐year MFS in group C1 and C2 were 0.69 versus 0.69 and 0.64, 0.64 respectively (*p* = 0.791); 3‐ and 5‐year OS were 0.76 versus 0.76 and 0.70 versus 0.70 respectively (*p* = 0.392). Kaplan Meier curves for OS by CINSARC prediction and treatment arm are reported in Figure 2.

#### 3.2.1 CINSARC prognostic value in Sarc‐LR and ‐HR

In Sarc‐LR, the 3 and 5‐year RFS in C1 and C2 were 0.69 versus 0.69 and 0.57 versus 0.57 respectively (*p* = 0.372); 3‐ and 5‐year MFS in group C1 and C2 were 0.79 versus 0.79 and 0.70 versus 0.66 respectively (*p* = 0.535); 3‐ and 5‐year OS were 0.85 versus 0.85 and 0.81 versus 0.78 respectively (*p* = 0.618).

In Sarc‐HR, the 3 and 5‐year RFS in C1 and C2 were 0.51 versus 0.34 and 0.57 versus 0.52 respectively (*p* = 0.811); 3 and 5‐year MFS in group C1 and C2 were 0.51 versus 0.34 and 0.62 versus 0.62 respectively (*p* = 0.508); 3‐ and 5‐year OS were 0.87 versus 0.70 and 0.78 versus 0.67 respectively (*p* = 0.806).

### 3.3 CINSARC predictive value: OS by CINSARC prediction and treatment arm

The 3‐ and 5‐year OS in C1 (*n* = 30) treated with ST and HT chemotherapy was 0.93 versus 0.84 and 0.76 versus 0.76 respectively (*p* = 0.251); 3‐ and 5‐year OS in C2 (*n* = 56) treated with ST and HT chemotherapy was 0.84 versus 0.72 and 0.70 versus 0.70 respectively (*p* = 0.349).

## 4 DISCUSSION

In this series of 86 high‐risk patients (based on age, histological subtype, FNCLCC grade, size and site) with localized STS treated with preoperative chemotherapy within a prospective, randomized, phase III study (ISG‐STS 1001 study), we did not observe any significant difference in RFS (*p* = 0.481), MFS (*p* = 0.740) and OS (*p* = 0.460) between CINSARC low‐risk (C1, *n* = 30) and high‐risk (C2, *n* = 56) patients. We also observed a similar OS both in C1 (*p* = 0.251) and C2 (*p* = 0.349) treated with ST versus HT chemotherapy.

A correlation between CINSARC, a transcriptional signature derived from the expression of 67 genes, and metastatic outcome was originally reported in 2010 in a cohort of 127 STS patients, the largest retrospective series analyzed so far. In that study patients scored as C1 and C2 showed a 5‐year MFS rate of 84% and 48%, respectively (*p* = 0.0005).^3^ The correlation was corroborated in additional retrospective studies on selected STS types, including GIST,^8^ synovial sarcoma,^6^ uterine leiomyosarcoma,^4^ STUMP^5^ and rhabdomyosarcoma.^7^

The ISG‐STS 1001 study is a prospective, randomized, phase III study including 287 patients affected by localized, high‐risk STS of the extremities or trunk wall and treated with 3 cycles of either full‐dose ST pre‐operative chemotherapy with epirubicin plus ifosfamide or HT chemotherapy. In this study there was a trend favoring ST both in RFS and OS, suggesting a degree of effectiveness of full dose epirubicin and ifosfamide.

Herein, we explored the possible prognostic value of CINSARC in this subgroup of localized, high‐risk STS patients treated within a prospective, randomized study with preoperative chemotherapy, by applying the signature in pre‐treatment tumor samples. Currently, there are no studies on CINSARC focusing on this population, which is of course only partially represented in the previously reported series. In this first prospective effort, we did not observe any relevant correlation between CINSARC groups and outcome, both in the whole population (RFS, *p* = 0.481; OS, *p* = 0.460) and in the subgroup of patients treated with ST (RFS, *p* = 0.623; OS, *p* = 0.391) or HT (RFS, *p* = 0.647; OS, *p* = 0.392) chemotherapy (see Figure 2).

This could certainly be due to the limited number of patients included in this study, which is its major limitation. The small sample size is due to technical issues, which restrained availability of samples for molecular investigations. When ISG‐STS 1001 study was started, back in 2011, CINSARC could only be performed on frozen untreated specimens, significantly narrowing the number of cases that could be tested. Although CINSARC was subsequently validated also on untreated archival material,^1^, ^2^ the competing presence of concomitant translational research efforts foreseen by the ISG‐STS 1001 trial, hampered the possibility to expand further the case series for this study. Of note, since all patients of the ISG‐STS 1001 trial were treated in the neoadjuvant setting, translational research projects had to rely on preoperative core needle biopsies.

Moreover, all patients included in the current series received preoperative chemotherapy, and data currently available show a potential benefit of neoadjuvant chemotherapy itself in high‐risk STS. Thus, it could be postulated that the activity of chemotherapy could somehow offset CINSARC prognostic value. However, in principle this could be true only of the subgroup of trial patients with a higher risk of death. In fact, a recently published post‐hoc observational study of the ISG‐STS 1001, using a cut‐off of 60% 10‐year predicted survival by the nomogram SARCULATOR, identified 2 subgroups of patients, with higher (Sarc‐HR) and lower risk (Sarc‐LR).^15^ In this study, neoadjuvant ST chemotherapy was superior to HT only in Sarc‐HR. In the same Sarc‐HR group, the observed 5‐year survival was superior than the predicted 5‐year survival in patients treated with ST chemotherapy. Such a difference was not detected in Sarc‐HR patients treated with HT chemotherapy, suggesting an effect of ST chemotherapy in this subgroup. Conversely, in the Sarc‐LR group, the reverse was seen.^15^ Indeed, when we explored CINSARC prognostic value in Sarc‐LR patients, we still did not observe any difference in RFS (*p* = 0.372), MFS (*p* = 0.535) or OS (*p* = 0.618) between C1 and C2 patients. In the Sarc‐HR patients treated with the least effective therapy, i.e. HT chemotherapy, the numbers are exceedingly low, but even here we would not see any difference between C1 and C2.

Finally, it is important to recall that CINSARC was derived by combining genes differentially expressed in FNCLCC grade 3 versus grades 1 and 2 and involved in mitosis control and chromosome integrity, in order to overcome the well‐known limitations of FNCLCC histological grading.^3^ However, we know today that, although histological grading remains a crucial prognostic factor in STS, histological type and tumor size can have a major impact on prognostication.^14^ These pathological and clinical features are of course not taken into account by CINSARC and could obviously explain the lack of a prognostic value in comparison to prognosticators valuing all these factors together, all the more in STS series including multiple histological types.

## 5 CONCLUSIONS

Our results suggest the hypothesis that in high‐risk, localized STS treated with neoadjuvant chemotherapy CINSARC may not separate patients with a different prognosis. Unfortunately, our sample was too low to reasonably narrow confidence intervals on all outcome indicators. Thus, it may just serve as hypothesis‐generating. The upcoming CHIC‐STS trial will only include G1–G2 patients, thus being outside this population, while more data will be provided by a prospective study currently ongoing at Fondazione IRCSS Istituto Nazionale Tumori (Milan) aiming at integrating radiomics, genomics and immune‐profiling into predictive and prognostic models in all STS patients (SARCOMICS). In general, further efforts on a larger scale validating the prognostic and predictive value of CINSARC in high‐risk STS patients, as enrolled in our neoadjuvant trial, are worthwhile.

## AUTHOR CONTRIBUTIONS

Anna Maria Frezza: conceptualization; data curation; formal analysis; investigation; methodology; project administration; resources; supervision; validation; writing original draft; review & editing. Silvia Stacchiotti: conceptualization; data curation; formal analysis; investigation; methodology; resources; supervision; validation; review & editing. Frederic Chibon: conceptualization; data curation; formal analysis; investigation; methodology; resources; supervision; validation; review & editing. Jean‐Michelle Coindre: data curation; investigation; methodology; supervision; validation; review & editing. Antoine Italiano: data curation; investigation; methodology; supervision; validation; review & editing. Cleofe Romagnosa: data curation; investigation; methodology; supervision; validation; review & editing. Silvia Bagué: data curation; investigation; methodology; supervision; validation; review & editing. Angelo Paolo Dei Tos: data curation; investigation; methodology; supervision; validation; review & editing. Luca Braglia: data curation; formal analysis; investigation; methodology; software; supervision; validation; writing original draft; review & editing. Emanuela Palmerini: data curation; investigation; methodology; supervision; validation; review & editing. Vittorio Quagliuolo: data curation; investigation; methodology; supervision; validation; review & editing. Javier Martin Broto: data curation; investigation; methodology; supervision; validation; review & editing. Antonio Lopez Pousa: data curation; investigation; methodology; supervision; validation; review & editing. Giovanni Grignani: data curation; investigation; methodology; supervision; validation; review & editing. Antonella Brunello: data curation; investigation; methodology; supervision; validation; review & editing. Jean‐Yves Blay: data curation; investigation; methodology; supervision; validation; review & editing. Robert Diaz Beveridge: data curation; investigation; methodology; supervision; validation; review & editing. Iwona Lugowska: data curation; investigation; methodology; supervision; validation; review & editing. Tom Lesluyes: conceptualization; data curation; investigation; methodology; supervision; validation; review & editing. Roberta Maestro: conceptualization; data curation; investigation; methodology; supervision; validation; writing original draft; review & editing. Franco Domenico Merlo: data curation; formal analysis; investigation; methodology; software; supervision; validation; writing original draft; review & editing. Paolo Giovanni Casali: conceptualization; data curation; investigation; methodology; supervision; validation; writing original draft; review & editing. Alessandro Gronchi: conceptualization; data curation; formal analysis; funding; investigation; methodology; esources; supervision; validation; writing original draft; review & editing.

## FUNDING INFORMATION

This work has been supported by Pharmamar.

## CONFLICT OF INTEREST

AMF has received institutional research funding from Advenchen, Amgen Dompè, Bayer, Daiichi Sankyo, Deciphera, Epizyme, Eli Lilly, Glaxo, Karyopharm, Novartis, Pfizer, Pharmamar, Springworks. PGC has received honoraria for participation in advisory board for Bayer, institutional research funding from Amgen Dompé, Advenchen, Bayer, Blueprint, Deciphera, Eli Lilly, Epizyme, Daiichi, GSK, Karyopharm, Novartis, Pfizer, PharmaMar, SpringWorks, AROG Pharmaceuticals and Eisai, non‐remunerated activities for the Italian Sarcoma Group, European School of Oncology, Federation of Italian Cooperative Groups and Rare Cancers Europe. GG has received research grants from Pharmamar, Novartis, Bayer and received honoraria for participation in advisory boards for Lilly, EISAI, Pharmamar, Novartis, Merck, Glaxo. EP has served on advisory boards for Amgen, Daiichi Sankyo, Lilly, Deciphera, Eusa Pharma, and SynOx Therapeutics has received other research support from Bristol Myers Squibb, Pfizer, PharmaMar, Daiichi Sankyo, Incyte, and travel support from Lilly, PharmaMar, and Takeda. SS has received consultancy fee or honoraria for participation in advisory board from Bavarian Nordic, Bayer, Daiichi Sankyo, Deciphera, Epizyme, Eli Lilly, Glaxo, Ikena, Maxivax, Novartis, Pharmamar; institutional research funding from Advenchen, Amgen Dompè, Bayer, Daiichi Sankyo, Deciphera, Epizyme, Eli Lilly, Glaxo, Karyopharm, Novartis, Pfizer, Pharmamar, Springworks. AB received consultancy fee or honoraria for participation in advisory board from Eli Lilly, Eisai, Glaxo‐Smith Kline; travel grants grants from PharmaMar and Ipsen. AG has received honoraria for participation in advisory boards for Novartis, Pfizer, Bayer, Lilly, PharmaMar, SpringWorks and Nanobiotix; invited speaker for Lilly, PharmaMar and research grant from PharmaMar. FC, JMC, AI, CR, SB, APDT, LB, VQ, JMB, ALS, JYB, RDB, IL, TL, RM, FDM, have nothing to declare.

## ETHICAL STATEMENT

The ISG‐STS 1001 study was approved by the institutional review board or ethics committee of each contributing institution and all patients included in the study signed a dedicated informed consent.

## Data Availability

The data that support the findings of this study are available on request from the corresponding author. The data are not publicly available due to privacy or ethical restrictions.
